# A SNP of miR‐146a is involved in bladder cancer relapse by affecting the function of bladder cancer stem cells via the miR‐146a signallings

**DOI:** 10.1111/jcmm.15480

**Published:** 2020-06-28

**Authors:** Tianen Wang, Yanfeng Yang, Zhiyong Wang, Xuechong Zhang, Dongsheng Li, Jinxing Wei

**Affiliations:** ^1^ Department of Urology The First Affiliated Hospital of Zhengzhou University Zhengzhou China

**Keywords:** BC stem cell, bladder cancer, miR‐146a, relapse, rs2910164

## Abstract

MiR‐146a‐5p in urine samples was recently reported to be possibly used as a prognostic marker for bladder cancer (BC). Interestingly, YAP1 and COX2 were both demonstrated to function as stem cell regulators in BC. Therefore, in this study, we aimed to establish the molecular mechanism underlying the role of miR‐146a, YAP1 and COX2 in BC relapse. We also studied the possibility of using the C > G genotype of miR‐146a rs2910164 SNP as an indicator of BC relapse. A total of 170 BC patients were assigned into different groups based on their genotypes of rs2910164 SNP. Kaplan‐Meier survival curves were plotted to compare the recurrence‐free rate among these groups. Real‐time PCR, Western Blot, bioinformatic analysis, luciferase assay and IHC assay were conducted to study the role of rs2910164 SNP in the progression of BC. Accordingly, GC/CC‐genotyped patients presented a higher risk of recurrence when compared with GG‐genotyped patients, while the expression of BC regulators was influenced by the presence of rs2910164. COX2 mRNA and YAP1 mRNA were, respectively, validated as direct target genes of miR‐146a, and the expression of YAP1 and COX2 mRNA/protein was both suppressed by miR‐146a precursors. The expression of ALDH1A1 mRNA/protein was inhibited upon the down‐regulation of YAP1, while the expression of let7 and SOX2 mRNA/protein was inhibited upon the down‐regulation of COX2. In conclusion, two signalling pathways, miR‐146a/YAP1/ALDH1A1 and miR‐146a/COX2/PGE2/let7/SOX2, were modulated by miR‐146a. As an SNP regulating the expression of miR‐146a, the rs2910164 G > C SNP could be utilized as a biomarker for BC relapse.

## 1 INTRODUCTION

As a malignancy of the urinary tract, bladder cancer (BC) causes about 170 000 deaths globally in 2012.^1^ The prevalence of BC increased significantly in recent years, because the systemic therapies used in the treatment of BC is hindered by drug resistance, tumour recurrence and metastasis.^2^ A past study showed that more than one third of BC patients suffered from intravesical tumour recurrence within 40 months after the treatment, and the grade of BC is associated with the susceptibility to tumour recurrence.^3^ After transurethral surgery of BC, the intravesical administration of mitomycin C and BCG significantly decreased the rate of BC recurrence.^4^ Past data also suggested that chemoresistance of BC can be caused by cancer stem cells (CSCs), whose proliferation after tumour resection may aid BC recurrence.^5^ Currently, CSCs are defined as tumour‐associated cells with self‐repair ability to render different tumour cell lineages heterogeneous.^6^

Sex‐determining region Y [SRY]‐box 2 (SOX2) can maintain the self‐repair and proliferation of CSCs in a wide range of human malignancies.^7^, ^8^ In addition, SOX2 also exerts a critical effect on paclitaxel‐ and gemcitabine‐induced drug resistance in several cancers.^9^, ^10^ It was shown that SOX2 is essential for chemoresistance to gemcitabine and cisplatin by acting as a key oncogene.^5^ Pityn´ski et al (2015) revealed that the age upon endometrial adenocarcinoma diagnosis is positively correlated with SOX2 expression, while a high level of SOX2 expression was closely related to improved survival in NMIBC patients.^11^ Similarly, YAP1 confers CSC traits and plays a protective role against chemotherapy‐induced apoptosis, thus providing a rationale for targeting the COX2/PGE2 and YAP1 signalling pathways to attenuate CSCs by uncovering the mechanism underlying COX2/PGE2‐induced CSC expansion and interaction with YAP1.^12^, ^13^, ^14^

MicroRNA (miRNA) is a type of small non‐coding RNA (ncRNA) with the function to negatively mediate the expression of its target genes. Therefore, miRNAs were shown to exert important effects on tumour progression and migration.^15^ Many studies have shown that miR‐200c affects the survival and growth of tumour cells by functioning as a tumour suppressor.^16^ It was shown that miR‐146a is essential for the maintenance of breast CSCs during EMT, potentially by repressing the expression of the Notch signalling inhibitor NUMB.^17^ So far, multiple biomarkers were shown to be correlated with BC recurrence.^18^ For example, the rs2910164 single nucleotide polymorphism (SNP) in miR‐146a is correlated with BC recurrence and can be used to predict BC prognosis.^19^ As a SNP located in pre‐miR‐146a, rs2910164 can affect the expression of miR‐146a, an inhibitor of BC proliferation.^19^, ^20^

A recent study by Sasaki H et al reported that the urinary level of microRNA‐146a‐5p (miR‐146a‐5p) was associated with the grade and invasion of BC, indicating that miR‐146a‐5p may be used as a prognostic marker for BC.^21^ In addition, the GG genotypes of SNP rs2910164 in miR‐146a have been reported to affect the risk and progression of gastric cancer.^22^ Meanwhile, YAP1 and COX2 were both demonstrated to function as stem cell regulators in BC.^12^, ^23^ Therefore, we aimed to establish the molecular mechanism underlying the role of miR‐146a, YAP1 and COX2 in BC relapse. Furthermore, we discussed the possibility of using miR‐146a rds2910164 C > G SNP as a biomarker for BC relapse.

## 2 MATERIALS AND METHODS

### 2.1 Human sample collection

In this study, a total of 170 patients aging from 36 to 78 (median: 51; mean: 45.65) who were diagnosed of BC were recruited to study the molecular mechanism underlying the role of miR‐146a, YAP1 and COX2 in BC progression and relapse. After their enrolment, peripheral blood samples were collected from each patient during fasting to measure the serum levels of miR‐146a, YAP1 and COX2. In addition, during the radical operation of BC, an appropriate amount of BC cancer tissues was collected from each patient to determine the expression of miR‐146a, YAP1 and COX2 as well as the genotypes of rs2910164 SNP in each sample. The experimental protocols for relevant mRNA and protein expression assays will be described in more detail in the following sections. Based on the genotypes of rs2910164 SNP, the patients were further grouped into a GG group (N = 92), a GC group (N = 62) and a CC group (N = 16). Subsequently, the clinical characteristics of the patients in different groups were compared. This study was approved by the ethics committee of our institution, and all participants have signed consent forms before the initiation of this study.

### 2.2 Genotyping by direct sequencing

The RNA content in each BC cancer tissue sample collected from the BC patients was isolated using TRIzol reagent (Invitrogen) following the recommendations on the manufacturer's manual. In the next step, the host DNA in each isolated RNA sample was eliminated by subjecting the sample to a DNase treatment with a Heat & Run kit (ArcticZymes), and the isolated RNA was then inactivated for 5 minutes by heating to 80°C. Subsequently, first‐strand cDNA corresponding to each RNA sample was synthesized using a Protoscript kit (New England Biolabs) following the recommendations on the kit manual. Finally, the miR‐146a gene in each sample was amplified by real‐time PCR and the genotypes of rs2910164 SNP located in miR‐146a were determined by direct sequencing using Illumina NextSeq 500 sequencer.

### 2.3 RNA isolation and real‐time PCR

Real‐time PCR was carried out in this study to assay the expression of miR‐146a, YAP1 mRNA, COX2 mRNA, ALDH1A1 mRNA, let7 and SOX2 mRNA in each clinical and cell sample. In this study, conventional methods of RNA isolation, RT reaction and quantitative PCR were used. In brief, total RNA and miRNA content were isolated from each sample using an miRNeasy Micro Kit (Qiagen) following the recommendations on the kit manual. In the next step, isolated miRNAs and mRNAs were reversely transcribed in cDNA templates using a miScript II RT kit (Qiagen) and a RevertAid RT Reverse Transcription kit (Thermo Fisher Scientific), respectively, following the instructions on the manuals of these kits. Then, real time was carried out using the synthesized cDNA templates and a SYBR Green Master Mix (ABI) on an ABI Prism 7900HT real‐time PCR machine (ABI) following the instructions of the manufacturer. Finally, the Ct values of disassociation curves were taken to quantify the relative expression of miR‐146a (Forward sequence: 5′‐GAGAACTGAATTCCATGG‐3′; Reverse sequence: 5′‐GAACATGTCTGCGTATCTC‐3′), YAP1 mRNA (Forward sequence: 5′‐TGTCCCAGATGAACGTCACAGC‐3′; Reverse sequence: 5′‐TGGTGGCTGTTTCACTGGAGCA‐3′), COX2 mRNA (Forward sequence: 5′‐CGGTGAAACTCTGGCTAGACAG‐3′; Reverse sequence: 5′‐GCAAACCGTAGATGCTCAGGGA‐3′), ALDH1A1 mRNA (Forward sequence: 5′‐CGGGAAAAGCAATCTGAAGAGGG‐3′; Reverse sequence: 5′‐GATGCGGCTATACAACACTGGC‐3′), let7 (Forward sequence: 5′‐TGAGGTAGTAGGTTGTATA‐3′; Reverse sequence: 5′‐GAACATGTCTGCGTATCTC‐3′) and SOX2 mRNA (Forward sequence: 5′‐GCTACAGCATGATGCAGGACCA‐3′; Reverse sequence: 5′‐TCTGCGAGCTGGTCATGGAGTT‐3′) in each sample using the 2^−ΔΔCt^ method. The thermocycling protocol for RT‑PCR was 10 minutes at 95˚C, followed by 40 cycles of 30 seconds at 95˚C, 30 seconds at 60˚C and 30 seconds at 72˚C, and the expression of U6 (Forward sequence: 5′‑CTCGCTTCGGCAGCACA‑3′; Reverse sequence: 5′‑AACGCTTCACGAATTTGCGT‑3′; for miR‐146a) and β‐actin (Forward sequence: 5′‑ CACCATTGGCAATGAGCGGTTC‑3′; Reverse sequence: 5′‑ AGGTCTTTGCGGATGTCCACGT‑3′) were used as the internal control.

### 2.4 Cell culture and transfection

T24 and RT4 cells were acquired from ATCC. In our laboratory, these cells were maintained in the DMEM medium (Gibco, Thermo Fisher Scientific) added with 10% foetal bovine serum (Gibco, Thermo Fisher Scientific). The cell incubation was carried out under 5% CO_2_ at 37°C. For transfection experiments, the cells were seeded into 24‐well plates and transfected with appropriate vectors using Lipofectamine 2000 (Invitrogen). The transfected cells were collected after 48 hours of transfection and were assayed for their expression of target genes.

### 2.5 Vector construction, mutagenesis and luciferase assay

In order to determine the regulatory relationship between miR‐146a and YAP1/COX2, a luciferase assay was carried out in this study using specifically designed luciferase vectors. In brief, the full 3′UTR sequences of YAP1/COX2 containing the binding sites for miR‐146a were amplified and subcloned into pcDNA vectors (Promega) to generate wild‐type vectors for YAP1 and COX2, respectively. In the next step, a QuickChange II XL Site‐Directed Mutagenesis Kit (Agilent) was utilized to introduce mutations into the 3′UTR sequences of YAP1/COX2 containing the binding sites for miR‐146a, respectively, and the mutant sequences were also subcloned into pcDNA vectors to generate mutant vectors for YAP1 and COX2, respectively. Then, T24 and RT4 cells were seeded into 24‐well plates and cotransfected with YAP1 or COX2 wild‐type/mutant vectors in conjunction with 30 nmol/L miR‐146a precursors, YAP1 siRNA or COX2 siRNA using Lipofectamine 2000. For the selection of concentrations, a concentration ladder at 30, 60 and 100 nmol/L of miRNA precursors or siRNAs was utilized. However, as the results of the transfection of miRNA precursors or siRNAs were all significant compared with the control while being comparable between different concentration groups, the lowest concentration, 30 nmol/L, was chosen in this study. The luciferase activity of transfected cells was assayed after 48 hours of transfection using a Bright‐Glo luciferase assay kit (Promega) following the kit manual on a Centro LB 960 luminometer (Berthold Technologies).

### 2.6 Western blot analysis

Tissue and cell samples were collected and rinsed two times using cold PBS (Gibco, Thermo Fisher Scientific). Then, the samples were lysed using a RIPA buffer containing appropriate protease inhibitors (Gibco, Thermo Fisher Scientific). The quality and quantity of protein isolated from each sample was measured using a DC protein assay (BioRad). Then, each isolated protein sample was separated using 10% SDS‐PAGE and subsequently blotted onto PVDF membranes (Thermo Fisher Scientific). After being incubated with anti‐YAP1 (dilution: 1:5000, cat.no. ab56701, Abcam), anti‐COX2 (dilution: 1:5000, cat.no. ab15191, Abcam, Cambridge, MA), anti‐ALDH1A1 (dilution: 1:5000, cat.no. ab227964, Abcam) and anti‐SOX2 (dilution: 1:5000, cat.no. ab97959, Abcam) primary antibodies and HRP‐tagged secondary antibodies (dilution: 1:10 000, cat.no. ab6759, Abcam) following a conventional protocol, the PVDF membranes were developed using an ECL kit (Thermo Fisher Scientific, Waltham, MA) and visualized using a Fusion‐Fx system (Vilber Lourmat) to determine the relative protein expression of YAP1, COX2, ALDH1A1 and SOX2. During the densitometric analysis, the expression of GAPDH was used as the internal control.

### 2.7 Immunohistochemistry assay

Tissue samples were routinely fixed, paraffin embedded, sliced to sections of 4 μm in thickness, incubated with 3% H_2_O_2_ (Sigma‐Aldrich) to remove endogenous peroxidase activity, incubated at 4°C overnight with anti‐YAP1 and anti‐COX2 primary antibodies (Abcam), washed by PBS, incubated at room temperature for 1 hour with secondary antibodies, coloured with DAB (Sigma‐Aldrich) and evaluated under a light microscope (Olympus).

### 2.8 Statistical analysis

All statistical analyses were carried out using Prism 7.0 (GraphPad). All data were represented using mean ± SD. Kaplan‐Meier recurrence‐free curves were plotted and analysed to compare the survival status among different groups. Two‐sided Student's *t* tests were used to compare the data of two groups, while one‐way ANOVA was used to compare the data of multiple groups.* P* < .05 was deemed statistically significant. All experiments were performed in triplicates.

## 3 RESULTS

### 3.1 Association between rs2910164 and the clinical characteristics of patients

In this study, a total of 170 BC patients were recruited. After collecting their blood and tissue samples for further genotyping analysis, these patients were further grouped into a GG group (N = 92), a GC group (N = 62) and a CC group (N = 16) based on their genotypes of rs2910164 polymorphism. As shown in Table 1, the clinical characteristics of GG‐genotyped BC patients and GC/CC‐genotyped BC patients showed no significant differences.

**TABLE 1:** Clinical characteristics of BC patients carrying different genotypes of rs2910164

Characteristics	GG (N = 92)	GC + CC (N = 78)	*P* value
Gender			
Male	75 (81.5)	65 (83.3)	.784
Female	17 (18.5)	13 (16.7)	
Age of diagnosis			
<65 y	35 (38.0)	28(36.0)	.847
≥65 y	57 (62.0)	50 (64.0)	
Family history of any other cancer			
Yes	13 (14.1)	12 (15.4)	.879
No	79 (85.9)	66 (84.6)	
Missing	0 (0.0)	0 (0.0)	
Smoking habit			
Smokers	34 (36.9)	30 (38.5)	.971
Non‐smokers	58 (63.1)	48 (61.5)	
Missing	0 (0.0)	0 (0.0)	
Years of smoking			
<20 y	58 (63.0)	51 (65.4)	.689
≥20 y	34 (37.0)	27 (34.6)	
Missing	0 (0.0)	0 (0.0)	
Cigarettes/day			
<10	62 (67.4)	54 (69.2)	.947
≥10	30 (32.6)	24 (30.8)	
Missing	0 (0.0)	0 (0.0)	
Drinking habit			
Drinkers	12 (13.0)	9 (11.5)	.887
Non‐drinkers	80 (87.0)	69 (88.5)	
Missing	0 (0.0)	0 (0.0)	
Years of drinking			
<30 y	80 (87.0)	66 (84.6)	.871
≥30 y	12 (13.0)	12 (15.4)	
Missing	0 (0.0)	0 (0.0)	

### 3.2 Association between rs2910164 and patient survival

**FIGURE 1:**
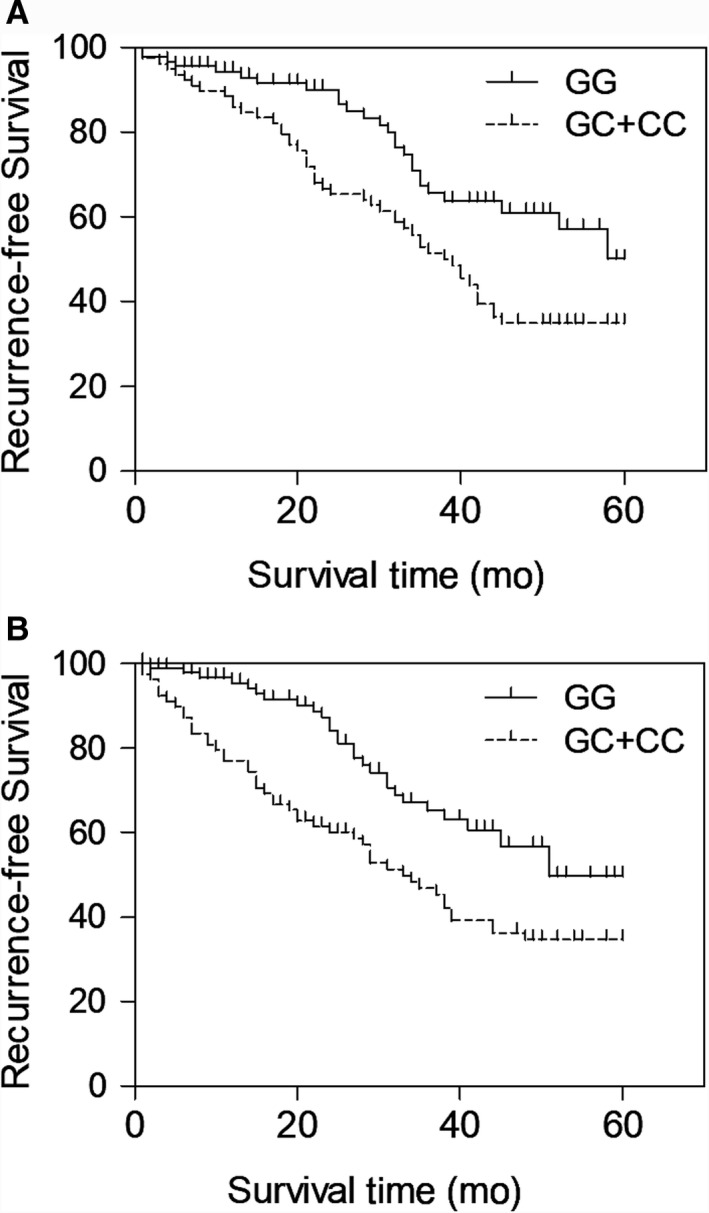
The association between rs2910164 and patient survival of BC patients carrying GG and GC + CC genotypes indicated by the Kaplan‐Meier recurrence‐free survival curves up to 60 months (A: blood samples; B: tissue samples)

In order to investigate the potential association between rs2910164 and patient survival, the recurrence‐free survival curves of up to 60 months were plotted. In the dominant genetic model shown in Figure 1, the BC patients carrying the C allele, that is GC/CC‐genotyped patients, presented a higher risk of recurrence than the patients carrying the G allele, that is GG‐genotyped patients. Therefore, the Kaplan‐Meier plots suggested that rs2910164 might play an essential role in the prognosis of BC.

### 3.3 Association between rs2910164 and BC‐related regulators

**FIGURE 2:**
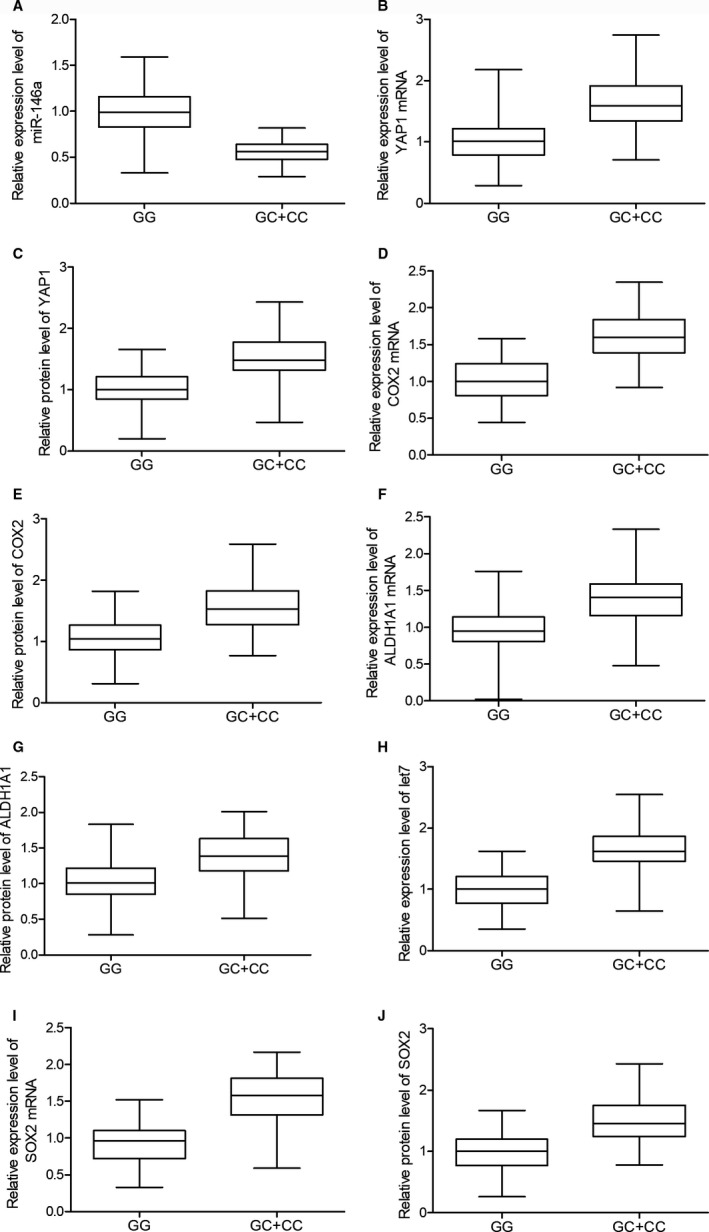
The association between rs2910164 and BC‐related regulators including miR‐146a (A), YAP1 mRNA (B), YAP1 protein (C), COX2 mRNA (D), COX2 protein (E), ALDH1A1 mRNA (F), ALDH1A1 protein (G), let7 (H), SOX2 mRNA (I) and SOX2 protein (J) was observed in blood samples collected from BC patients carrying GG and GC + CC genotypes. The dysregulation of the expressions of BC‐related regulators was observed in different patient groups

**FIGURE 3:**
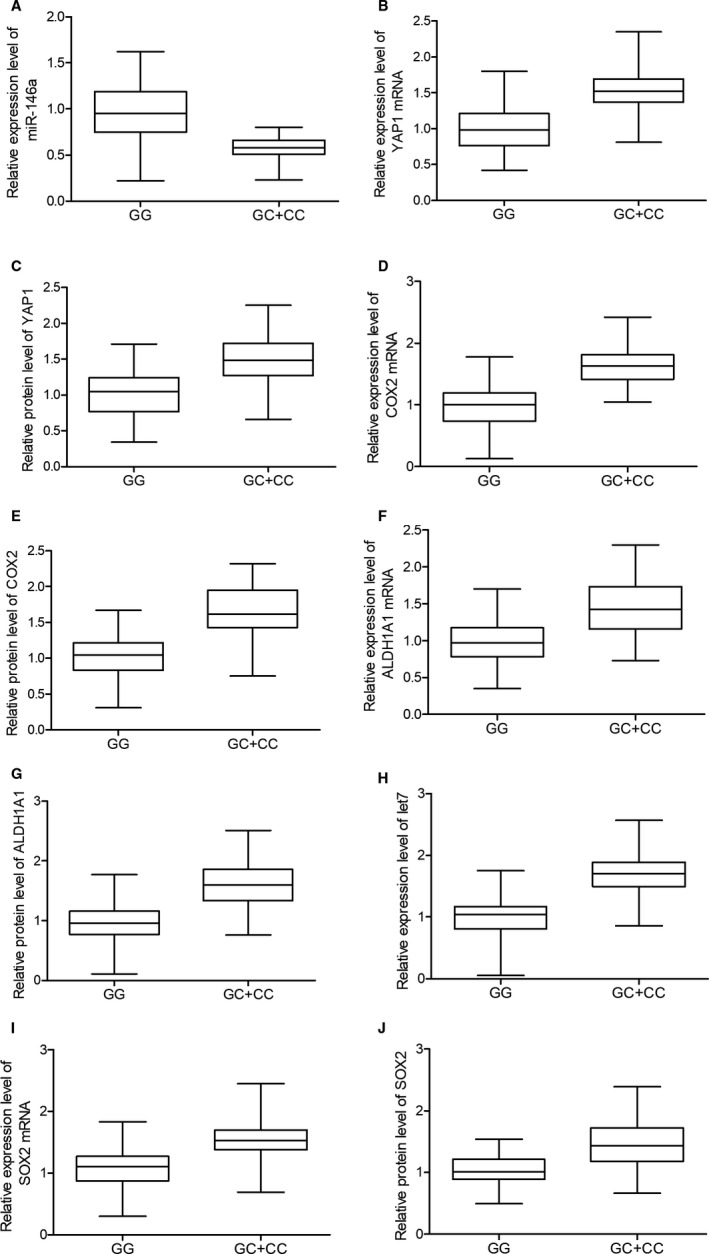
The expression of BC‐related regulators including miR‐146a (A), YAP1 mRNA (B), YAP1 protein (C), COX2 mRNA (D), COX2 protein (E), ALDH1A1 mRNA (F), ALDH1A1 protein (G), let7 (H), SOX2 mRNA (I) and SOX2 protein (J) was observed in tissue samples collected from BC patients carrying GG and GC + CC genotypes to study the association between rs2910164 and BC‐related regulators

**FIGURE 4:**
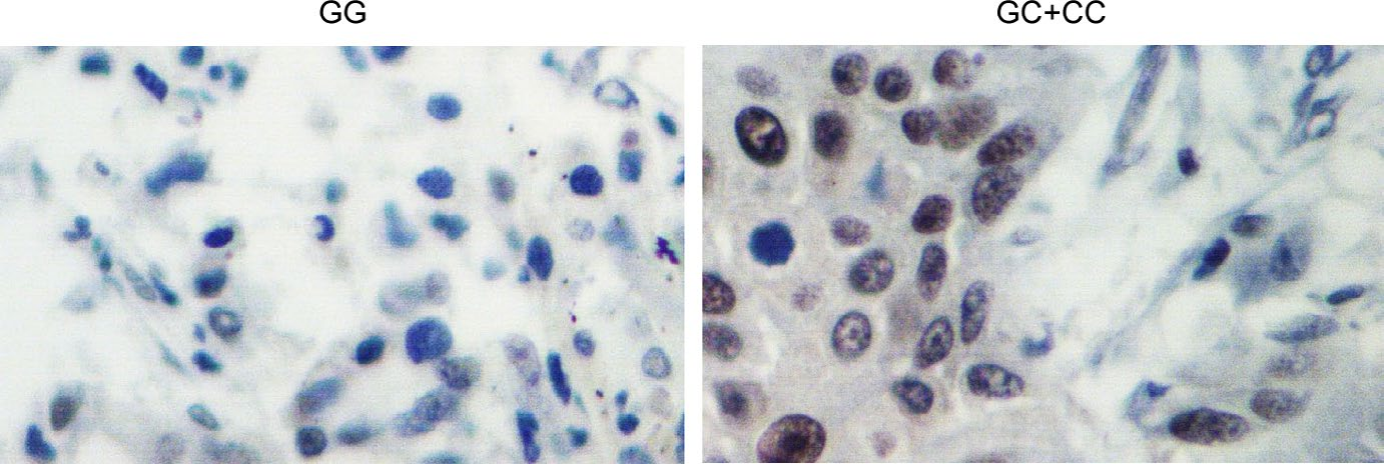
The YAP1 protein expression in tissue samples collected from patients carrying GG and GC + CC genotypes was shown to be significantly decreased GG‐genotyped patients by IHC assay

**FIGURE 5:**
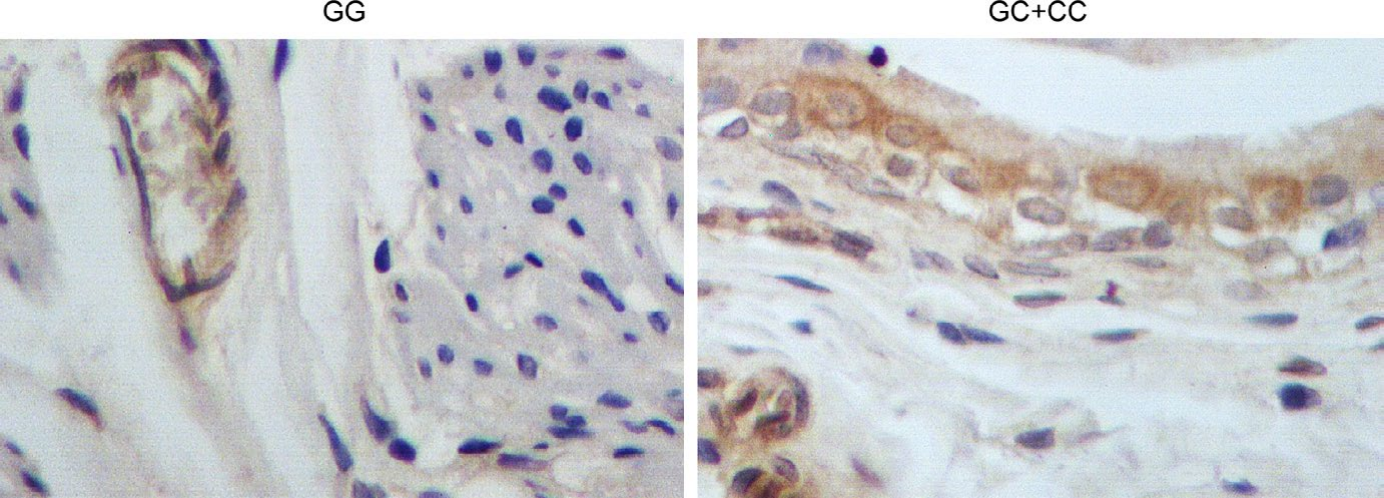
The IHC results of COX2 protein expression in tissue samples collected from patients carrying GG and GC + CC genotypes showed an evident down‐regulation of COX2 protein in the presence of GG homozygote in BC patients

The expression levels of possible regulators of BC, including miR‐146a, YAP1 mRNA/protein, COX2 mRNA/protein, ALDH1A1 mRNA/protein, let7 and SOX2 mRNA/protein, were compared among the blood and tissue samples grouped according to the genotypes of the patients. As shown in Figure 2, miR‐146a (Figure 2A) was evidently up‐regulated while the expression of YAP1 mRNA/protein (Figure 2B,C) and COX2 mRNA/protein (Figure 2D,E) was both down‐regulated in blood samples collected from GG‐genotyped patients compared with that in GC/CC‐genotyped patients. Meanwhile, the expression of ALDH1A1 mRNA/protein (Figure 2F,G), let7 (Figure 2H) and SOX2 mRNA/protein (Figure 2I,J) was all significantly decreased in the presence of GG homozygote in BC patients. Similar results were observed in tissue samples (Figure 3). Furthermore, in the IHC assay of tumour tissues, the expression of YAP1 (Figure 4) and COX2 (Figure 5) proteins was significantly higher in the GC + CC group compared with that in the GG group. Therefore, it can be concluded that rs2910164 is associated with the expression of several BC‐related regulators possibly via regulating the expression of miR‐146a.

### 3.4 Association between miR‐146a and COX2/YAP1 mRNA

**FIGURE 6:**
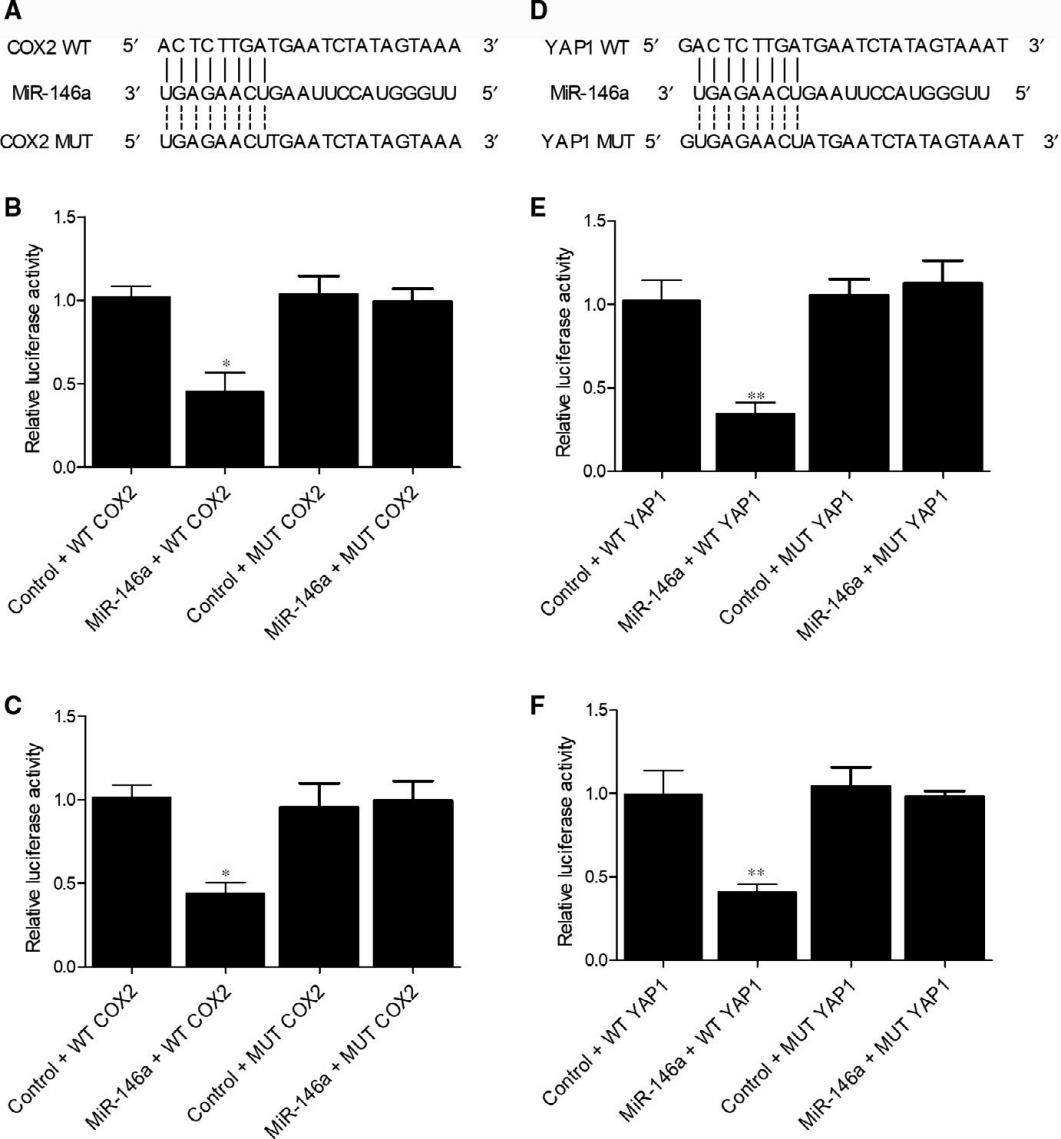
The association between miR‐146a and COX2 was identified by the putative binding site on 3’UTR of COX2 mRNA (A) and the evidently decreased relative luciferase activity of wild‐type COX2 mRNA in T24 cells (B) or RT4 cells (C) transfected with miR‐146a; moreover, YAP1 mRNA was validated to be a target gene of miR‐146a by detecting a potential binding site of on 3’UTR of YAP1 mRNA (D) and the suppressed relative luciferase activity of wild‐type YAP1 mRNA in T24 cells (E) or RT4 cells (F) transfected with miR‐146a (*P value < 0.01 vs control + WT COX2 group; **P value < .01 vs control + WT YAP1 group; WT, wild‐type; MUT, mutant)

In order to clarify the regulatory relationship between miR‐146a and BC‐related regulators, online bioinformatic tools (www.targetscan.org and www.mirdb.org) were utilized to identify the location of miR‐146a binding sites on the 3’UTR of COX2 (Figure 6A) and YAP1 (Figure 6D) mRNA, respectively. In the subsequent luciferase assays, the relative luciferase activity of wild‐type COX2 mRNA was significantly suppressed by the transfection of miR‐146a in T24 (Figure 6B) and RT4 (Figure 6C) cells. In contrary, the relative luciferase activity of mutant COX2 mRNA was not influenced by the transfection with miR‐146a or control miRNAs, thus validating the role of COX2 mRNA as a direct target gene of miR‐146a. Moreover, the relative luciferase activity of wild‐type YAP1 mRNA was decreased in the presence of miR‐146a in T24 (Figure 6E) and RT4 (Figure 6F) cells. Therefore, YAP1 mRNA was also validated as a target gene of miR‐146a. Altogether, two possible signalling pathways were identified, that is the miR‐146a/COX2 mRNA and the miR‐146a/YAP1 mRNA signalling pathways.

### 3.5 Association between miR‐146a signalling and BC

**FIGURE 7:**
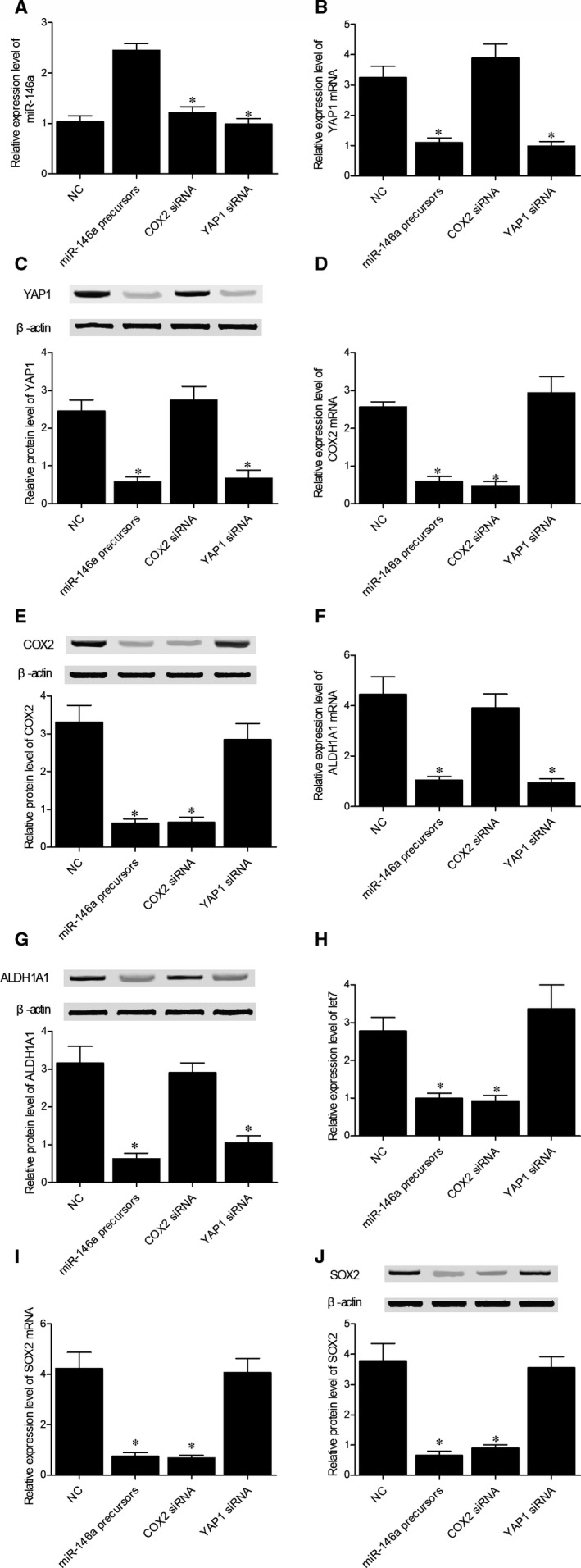
The association between miR‐146a signalling and BC in T24 cells was investigated via observing the different expressions of miR‐146a (A), YAP1 mRNA (B), YAP1 protein (C), COX2 mRNA (D), COX2 protein (E), ALDH1A1 mRNA (F), ALDH1A1 protein (G), let‐7 (H), SOX2 mRNA (I) and SOX2 protein (J) in T24 cells, respectively, transfected with negative controls, miR‐146a precursors, COX2 siRNA or YAP1 siRNA (*P value < .01 vs NC group; NC, negative control)

**FIGURE 8:**
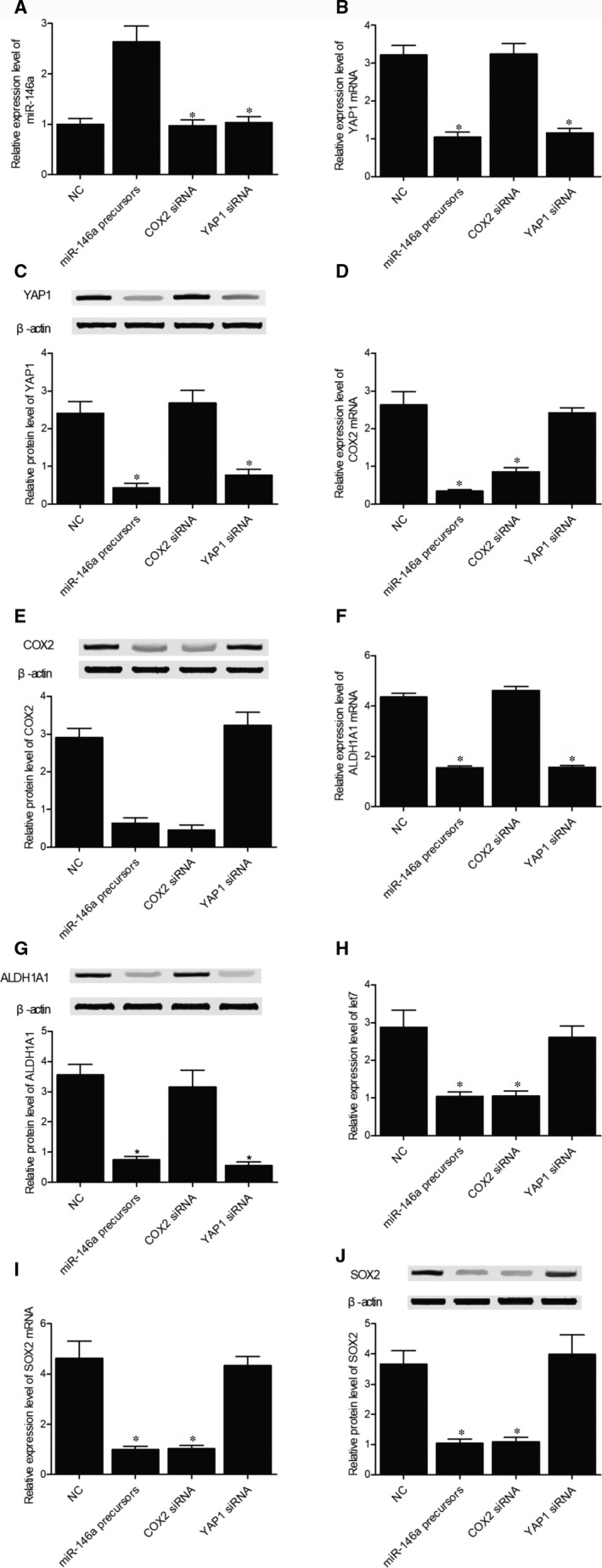
The expressions of BC‐related regulators including miR‐146a (A), YAP1 mRNA (B), YAP1 protein (C), COX2 mRNA (D), COX2 protein (E), ALDH1A1 mRNA (F), ALDH1A1 protein (G), let‐7 (H), SOX2 mRNA (I) and SOX2 protein (J) were observed in RT4 cells transfected with negative controls, miR‐146a precursors, COX2 siRNA or YAP1 siRNA to study the association between miR‐146a signalling and BC in RT4 cells (*P value < .01 vs NC group; NC, negative control)

To further investigate the role of rs2910164 and miR‐146a signalling in BC, T24 cells were transfected with miR‐146a precursors, COX2 siRNA, YAP1 siRNA or a negative control. As shown in Figure 7, the level of miR‐146a (Figure 7A) was significantly increased with the transfection of miR‐146a precursors. Moreover, YAP1 mRNA/protein (Figure 7B,C) expression was both suppressed in the presence of miR‐146a precursors and YAP1 siRNA, while miR‐146a precursors and COX2 siRNA both suppressed the expression of COX2 mRNA/protein (Figure 7D,E). Therefore, it can be concluded that the up‐regulation of miR‐146a leads to the down‐regulated expression of its target genes YAP1 and COX2. Meanwhile, both miR‐146a precursors and YAP1 siRNA inhibited the relative expression of ALDH1A1 mRNA/protein (Figure 7F,G) in T24 cells. Furthermore, the expression of let7 (Figure 7H) and SOX2 mRNA/protein (Figure 7I,J) was down‐regulated in the presence of miR‐146a precursors and COX2 siRNA in T24 cells. Same results were also obtained in RT4 cells (Figure 8). Therefore, it can be concluded that the miR‐146a/YAP1/ALDH1A1 and miR‐146a/COX2/PGE2/let7/SOX2 signalling pathways are regulated by rs2910164 via the expression of miR‐146a. As a result, the rs2910164 G > C SNP may be utilized as a biomarker for BC relapse.

## 4 DISCUSSION

As the most frequently observed genetic mutations in miRNAs and mRNAs, SNPs were found to participate in tumorigenesis.^24^ Located in the precursor of miR‐146a, the rs2910164 SNP was shown to regulate the expression of mature miR‐146a and hence was correlated with the susceptibility to many cancers.^25^, ^26^ Interestingly, a recent study investigated the correlation between BC and some SNPs located in miRNAs, although the role of these SNPs still remains controversial.^19^, ^27^ In this study, GC/CC‐genotyped patients showed a higher risk of BC recurrence compared with the patients carrying the G allele. In addition, a total of 170 BC patients in this study were grouped into the GG group (N = 92), GC group (N = 62) and CC group (N = 16) based on their genotypes of rs2910164 polymorphism, but no significant differences were observed among different groups in term of their clinical characteristics.

In addition, miR‐146a expression is increased by Snail in the stem cells of colon cancer and breast cancer.^28^, ^29^ However, the function of miR‐146a/b in haematopoietic cells remains unclear, although it was shown that the expression of miR‐146a is high in BM CD34 + HSPC cells.^30^ In this study, COX2 mRNA and YAP1 mRNA were both validated as direct target genes of miR‐146a, and the expression of YAP1 mRNA/protein and COX2 mRNA/protein was both suppressed in the presence of miR‐146a precursors. Moreover, the expression of ALDH1A1 mRNA/protein was inhibited upon the down‐regulation of YAP1 expression, while the expression of let7 and SOX2 mRNA/protein was inhibited upon the down‐regulation of COX2 expression. In addition, miR‐146a expression was evidently up‐regulated while the mRNA/protein expression of YAP1 and COX2 was both down‐regulated in GG‐genotyped patients compared with that in GC/CC‐genotyped patients, and the expression of let7, ALDH1A1 mRNA/protein and SOX2 mRNA/protein was all significantly decreased in patients carrying the GG homozygote.

Recently, multiple miRNAs, including miR‐146a, miR‐16, miR‐137, miR‐26b and miR‐101, were found to regulate the expression of COX‐2.^31^ Moreover, miR‐146a can reduce inflammatory response via NFκB signalling.^32^ As a gene whose expression is activated by multiple stimuli, COX2 was shown to affect the progression of COPD.^33^ In addition, COX2 increases the level of PGE2 in the body by accelerating the synthesis of PGE2 from fibroblasts in COPD patients.^34^ An elevated level of LIN28 was found to reduce the expression of let‐7 miRNA, a key promoter of cell proliferation and differentiation.^35^ It was shown that by promoting the expression of LIN‐28 via β‐catenin and SOX2 signalling, miR‐208a could inhibit let‐7a expression.^36^ SOX2 and Yes‐associated protein1 (YAP1) were studied for their possible correlation with CSC traits. As a key transcription factor, SOX2 enhances the pluripotency of stem cells.^37^ In skin squamous cell cancer, lung cancer, oesophageal cancer and medulloblastoma, SOX2 is essential for tumour occurrence and progression by regulating the expression of genes involved in the invasion, survival and proliferation of CSC.^38^ As a SOX family member, SOX2 plays important roles in determining cell fate.^39^ Moreover, the abnormal SOX2 expression was observed in multiple cancers.^40^ In particular, the expression of SOX2 is correlated with the prognosis and progression of BC.^41^ It was shown that as a biomarker of CSCs in BC, Sox2 can be used as a target in the treatment of BC.^42^ Furthermore, Zhang et al showed that the expression of Sox2 was correlated with the prognosis of ovarian cancer in human.^43^ Additionally, high expression of Sox2 is present in a large proportion of patients with an advanced neuroendocrine cancer.^44^ Moreover, Sox2 expression in advanced oesophageal squamous cell cancer is apparently increased.^45^ It was also shown that the expression of Sox2 is correlated with the risk of BC recurrence.^11^

YAP1 is a downstream transcription coactivator of the Hippo signalling and can regulate the activity of TEAD to affect the proliferation of stem cells.^46^ Moreover, the expression of YAP1 in the context of SOX2, OCT4 and KLF4 promotes iPSC reprogramming, indicating that YAP1 is a key regulator for the stem cell pluripotency.^47^ Similarly, YAP1 confers CSC traits and plays a protective role against chemotherapy‐induced apoptosis.^14^, ^46^ However, the induction of SOX2 could not completely restore cancer stem cell properties attenuated by the suppression of COX2 and YAP1, raising the possibility that YAP1 and COX2/PGE2 signalling as well as other tumorigenic pathways may also contribute to the maintenance of SOX2‐independent CSCs along with an aggressive tumour behaviour.^12^, ^46^ It was shown that the COX2/PGE2 and YAP1 signalling pathways work jointly to regulate the activity of urothelial CSCs, and the activation of these pathways reduces the efficacy of systemic therapy by expanding CSCs.^12^

## 5 CONCLUSION

We suggested for the first time that rs2910164 C > G of miR‐146a is involved in BC relapse via the miR‐146a/YAP1 and miR‐146a/COX2/PGE2/let‐7a/SOX2 signalling pathways. In addition, the G allele of rs2910164 enhanced miR‐146a expression while inhibiting the expression of YAP1 and COX2. Additionally, COX2 is responsible for the production of PGE2, while the down‐regulation of PGE2 expression also reduced SOX2 expression. Finally, YAP1 and SOX2 are implicated in BC relapse, suggesting that miR‐146a rs2910164 C > G SNP may be used as a biomarker for BC relapse.

## CONFLICT OF INTEREST

None.

## AUTHORS' CONTRIBUTIONS

TW planed the study, YY and ZW collected the literature, TW, YY, ZW and XZ collected the data, DL and JW analysed and visualized the data, TW and YY drafted the manuscript and all the other co‐authors approved the final manuscript.

## ETHICAL STATEMENT

This study was approved by the ethics committee of our institution.

## CONSENT STATEMENT

All participants have signed consent forms before the initiation of this study.

## Data Availability

The data that support the findings of this study are available from the corresponding author upon reasonable request.
